# Supplementary material for: An open-source framework for large-scale, flexible evaluation of biomedical text mining systems
Source: J Biomed Discov Collab. 2008 Jan 29;3:1. doi: 10.1186/1747-5333-3-1 (PMC2276192; doi:10.1186/1747-5333-3-1)
Supplement: Additional file 1 — Evaluation platform source code. The additional file contains the software evaluation framework discussed in this paper. The most current release can be downloaded from SourceForge.net [1]. See the accompanying README file for instructions on installation and use. Also included in the distribution is a collection of UIMA wrappers for some commonly used BioNLP tools and annotated corpora. [file 1747-5333-3-1-S1.zip › bionlp_uima_repository-1.0.1/docs/api/src-html/edu/uchsc/ccp/uima/annotation/CCPTextAnnotation_Type.html]

```
001    
002    /* First created by JCasGen Tue Aug 28 16:44:10 MDT 2007 */
003    package edu.uchsc.ccp.uima.annotation;
004    
005    import org.apache.uima.cas.Feature;
006    import org.apache.uima.cas.FeatureStructure;
007    import org.apache.uima.cas.Type;
008    import org.apache.uima.cas.impl.CASImpl;
009    import org.apache.uima.cas.impl.FSGenerator;
010    import org.apache.uima.cas.impl.FeatureImpl;
011    import org.apache.uima.cas.impl.TypeImpl;
012    import org.apache.uima.jcas.JCas;
013    import org.apache.uima.jcas.JCasRegistry;
014    
015    /** The CCP TextAnnotation extends the base annotation class to include an annotation ID, the capability for multiple annotation spans, a link to the annotator responsible for generating the annotation, membership to annotation sets, and a link to a class mention which defines the class of this annotation.
016     * Updated by JCasGen Tue Aug 28 16:44:10 MDT 2007
017     * @generated */
018    public class CCPTextAnnotation_Type extends CCPAnnotation_Type {
019      /** @generated */
020      protected FSGenerator getFSGenerator() {return fsGenerator;}
021      /** @generated */
022      private final FSGenerator fsGenerator = 
023        new FSGenerator() {
024          public FeatureStructure createFS(int addr, CASImpl cas) {
025                             if (CCPTextAnnotation_Type.this.useExistingInstance) {
026                               // Return eq fs instance if already created
027                         FeatureStructure fs = CCPTextAnnotation_Type.this.jcas.getJfsFromCaddr(addr);
028                         if (null == fs) {
029                           fs = new CCPTextAnnotation(addr, CCPTextAnnotation_Type.this);
030                               CCPTextAnnotation_Type.this.jcas.putJfsFromCaddr(addr, fs);
031                               return fs;
032                         }
033                         return fs;
034            } else return new CCPTextAnnotation(addr, CCPTextAnnotation_Type.this);
035              }
036        };
037      /** @generated */
038      public final static int typeIndexID = CCPTextAnnotation.typeIndexID;
039      /** @generated 
040         @modifiable */
041      public final static boolean featOkTst = JCasRegistry.getFeatOkTst("edu.uchsc.ccp.uima.annotation.CCPTextAnnotation");
042     
043      /** @generated */
044      final Feature casFeat_annotationID;
045      /** @generated */
046      final int     casFeatCode_annotationID;
047      /** @generated */ 
048      public int getAnnotationID(int addr) {
049            if (featOkTst && casFeat_annotationID == null)
050          jcas.throwFeatMissing("annotationID", "edu.uchsc.ccp.uima.annotation.CCPTextAnnotation");
051        return ll_cas.ll_getIntValue(addr, casFeatCode_annotationID);
052      }
053      /** @generated */    
054      public void setAnnotationID(int addr, int v) {
055            if (featOkTst && casFeat_annotationID == null)
056          jcas.throwFeatMissing("annotationID", "edu.uchsc.ccp.uima.annotation.CCPTextAnnotation");
057        ll_cas.ll_setIntValue(addr, casFeatCode_annotationID, v);}
058        
059      
060     
061      /** @generated */
062      final Feature casFeat_annotator;
063      /** @generated */
064      final int     casFeatCode_annotator;
065      /** @generated */ 
066      public int getAnnotator(int addr) {
067            if (featOkTst && casFeat_annotator == null)
068          jcas.throwFeatMissing("annotator", "edu.uchsc.ccp.uima.annotation.CCPTextAnnotation");
069        return ll_cas.ll_getRefValue(addr, casFeatCode_annotator);
070      }
071      /** @generated */    
072      public void setAnnotator(int addr, int v) {
073            if (featOkTst && casFeat_annotator == null)
074          jcas.throwFeatMissing("annotator", "edu.uchsc.ccp.uima.annotation.CCPTextAnnotation");
075        ll_cas.ll_setRefValue(addr, casFeatCode_annotator, v);}
076        
077      
078     
079      /** @generated */
080      final Feature casFeat_documentSectionID;
081      /** @generated */
082      final int     casFeatCode_documentSectionID;
083      /** @generated */ 
084      public int getDocumentSectionID(int addr) {
085            if (featOkTst && casFeat_documentSectionID == null)
086          jcas.throwFeatMissing("documentSectionID", "edu.uchsc.ccp.uima.annotation.CCPTextAnnotation");
087        return ll_cas.ll_getIntValue(addr, casFeatCode_documentSectionID);
088      }
089      /** @generated */    
090      public void setDocumentSectionID(int addr, int v) {
091            if (featOkTst && casFeat_documentSectionID == null)
092          jcas.throwFeatMissing("documentSectionID", "edu.uchsc.ccp.uima.annotation.CCPTextAnnotation");
093        ll_cas.ll_setIntValue(addr, casFeatCode_documentSectionID, v);}
094        
095      
096     
097      /** @generated */
098      final Feature casFeat_annotationSets;
099      /** @generated */
100      final int     casFeatCode_annotationSets;
101      /** @generated */ 
102      public int getAnnotationSets(int addr) {
103            if (featOkTst && casFeat_annotationSets == null)
104          jcas.throwFeatMissing("annotationSets", "edu.uchsc.ccp.uima.annotation.CCPTextAnnotation");
105        return ll_cas.ll_getRefValue(addr, casFeatCode_annotationSets);
106      }
107      /** @generated */    
108      public void setAnnotationSets(int addr, int v) {
109            if (featOkTst && casFeat_annotationSets == null)
110          jcas.throwFeatMissing("annotationSets", "edu.uchsc.ccp.uima.annotation.CCPTextAnnotation");
111        ll_cas.ll_setRefValue(addr, casFeatCode_annotationSets, v);}
112        
113       /** @generated */
114      public int getAnnotationSets(int addr, int i) {
115            if (featOkTst && casFeat_annotationSets == null)
116          jcas.throwFeatMissing("annotationSets", "edu.uchsc.ccp.uima.annotation.CCPTextAnnotation");
117        if (lowLevelTypeChecks)
118          return ll_cas.ll_getRefArrayValue(ll_cas.ll_getRefValue(addr, casFeatCode_annotationSets), i, true);
119        jcas.checkArrayBounds(ll_cas.ll_getRefValue(addr, casFeatCode_annotationSets), i);
120            return ll_cas.ll_getRefArrayValue(ll_cas.ll_getRefValue(addr, casFeatCode_annotationSets), i);
121      }
122       
123      /** @generated */ 
124      public void setAnnotationSets(int addr, int i, int v) {
125            if (featOkTst && casFeat_annotationSets == null)
126          jcas.throwFeatMissing("annotationSets", "edu.uchsc.ccp.uima.annotation.CCPTextAnnotation");
127        if (lowLevelTypeChecks)
128          ll_cas.ll_setRefArrayValue(ll_cas.ll_getRefValue(addr, casFeatCode_annotationSets), i, v, true);
129        jcas.checkArrayBounds(ll_cas.ll_getRefValue(addr, casFeatCode_annotationSets), i);
130        ll_cas.ll_setRefArrayValue(ll_cas.ll_getRefValue(addr, casFeatCode_annotationSets), i, v);
131      }
132     
133     
134      /** @generated */
135      final Feature casFeat_numberOfSpans;
136      /** @generated */
137      final int     casFeatCode_numberOfSpans;
138      /** @generated */ 
139      public int getNumberOfSpans(int addr) {
140            if (featOkTst && casFeat_numberOfSpans == null)
141          jcas.throwFeatMissing("numberOfSpans", "edu.uchsc.ccp.uima.annotation.CCPTextAnnotation");
142        return ll_cas.ll_getIntValue(addr, casFeatCode_numberOfSpans);
143      }
144      /** @generated */    
145      public void setNumberOfSpans(int addr, int v) {
146            if (featOkTst && casFeat_numberOfSpans == null)
147          jcas.throwFeatMissing("numberOfSpans", "edu.uchsc.ccp.uima.annotation.CCPTextAnnotation");
148        ll_cas.ll_setIntValue(addr, casFeatCode_numberOfSpans, v);}
149        
150      
151     
152      /** @generated */
153      final Feature casFeat_spans;
154      /** @generated */
155      final int     casFeatCode_spans;
156      /** @generated */ 
157      public int getSpans(int addr) {
158            if (featOkTst && casFeat_spans == null)
159          jcas.throwFeatMissing("spans", "edu.uchsc.ccp.uima.annotation.CCPTextAnnotation");
160        return ll_cas.ll_getRefValue(addr, casFeatCode_spans);
161      }
162      /** @generated */    
163      public void setSpans(int addr, int v) {
164            if (featOkTst && casFeat_spans == null)
165          jcas.throwFeatMissing("spans", "edu.uchsc.ccp.uima.annotation.CCPTextAnnotation");
166        ll_cas.ll_setRefValue(addr, casFeatCode_spans, v);}
167        
168       /** @generated */
169      public int getSpans(int addr, int i) {
170            if (featOkTst && casFeat_spans == null)
171          jcas.throwFeatMissing("spans", "edu.uchsc.ccp.uima.annotation.CCPTextAnnotation");
172        if (lowLevelTypeChecks)
173          return ll_cas.ll_getRefArrayValue(ll_cas.ll_getRefValue(addr, casFeatCode_spans), i, true);
174        jcas.checkArrayBounds(ll_cas.ll_getRefValue(addr, casFeatCode_spans), i);
175            return ll_cas.ll_getRefArrayValue(ll_cas.ll_getRefValue(addr, casFeatCode_spans), i);
176      }
177       
178      /** @generated */ 
179      public void setSpans(int addr, int i, int v) {
180            if (featOkTst && casFeat_spans == null)
181          jcas.throwFeatMissing("spans", "edu.uchsc.ccp.uima.annotation.CCPTextAnnotation");
182        if (lowLevelTypeChecks)
183          ll_cas.ll_setRefArrayValue(ll_cas.ll_getRefValue(addr, casFeatCode_spans), i, v, true);
184        jcas.checkArrayBounds(ll_cas.ll_getRefValue(addr, casFeatCode_spans), i);
185        ll_cas.ll_setRefArrayValue(ll_cas.ll_getRefValue(addr, casFeatCode_spans), i, v);
186      }
187     
188     
189      /** @generated */
190      final Feature casFeat_classMention;
191      /** @generated */
192      final int     casFeatCode_classMention;
193      /** @generated */ 
194      public int getClassMention(int addr) {
195            if (featOkTst && casFeat_classMention == null)
196          jcas.throwFeatMissing("classMention", "edu.uchsc.ccp.uima.annotation.CCPTextAnnotation");
197        return ll_cas.ll_getRefValue(addr, casFeatCode_classMention);
198      }
199      /** @generated */    
200      public void setClassMention(int addr, int v) {
201            if (featOkTst && casFeat_classMention == null)
202          jcas.throwFeatMissing("classMention", "edu.uchsc.ccp.uima.annotation.CCPTextAnnotation");
203        ll_cas.ll_setRefValue(addr, casFeatCode_classMention, v);}
204        
205      
206    
207    
208    
209      /** initialize variables to correspond with Cas Type and Features
210            * @generated */
211      public CCPTextAnnotation_Type(JCas jcas, Type casType) {
212        super(jcas, casType);
213        casImpl.getFSClassRegistry().addGeneratorForType((TypeImpl)this.casType, getFSGenerator());
214    
215     
216        casFeat_annotationID = jcas.getRequiredFeatureDE(casType, "annotationID", "uima.cas.Integer", featOkTst);
217        casFeatCode_annotationID  = (null == casFeat_annotationID) ? JCas.INVALID_FEATURE_CODE : ((FeatureImpl)casFeat_annotationID).getCode();
218    
219     
220        casFeat_annotator = jcas.getRequiredFeatureDE(casType, "annotator", "edu.uchsc.ccp.uima.annotation.CCPAnnotator", featOkTst);
221        casFeatCode_annotator  = (null == casFeat_annotator) ? JCas.INVALID_FEATURE_CODE : ((FeatureImpl)casFeat_annotator).getCode();
222    
223     
224        casFeat_documentSectionID = jcas.getRequiredFeatureDE(casType, "documentSectionID", "uima.cas.Integer", featOkTst);
225        casFeatCode_documentSectionID  = (null == casFeat_documentSectionID) ? JCas.INVALID_FEATURE_CODE : ((FeatureImpl)casFeat_documentSectionID).getCode();
226    
227     
228        casFeat_annotationSets = jcas.getRequiredFeatureDE(casType, "annotationSets", "uima.cas.FSArray", featOkTst);
229        casFeatCode_annotationSets  = (null == casFeat_annotationSets) ? JCas.INVALID_FEATURE_CODE : ((FeatureImpl)casFeat_annotationSets).getCode();
230    
231     
232        casFeat_numberOfSpans = jcas.getRequiredFeatureDE(casType, "numberOfSpans", "uima.cas.Integer", featOkTst);
233        casFeatCode_numberOfSpans  = (null == casFeat_numberOfSpans) ? JCas.INVALID_FEATURE_CODE : ((FeatureImpl)casFeat_numberOfSpans).getCode();
234    
235     
236        casFeat_spans = jcas.getRequiredFeatureDE(casType, "spans", "uima.cas.FSArray", featOkTst);
237        casFeatCode_spans  = (null == casFeat_spans) ? JCas.INVALID_FEATURE_CODE : ((FeatureImpl)casFeat_spans).getCode();
238    
239     
240        casFeat_classMention = jcas.getRequiredFeatureDE(casType, "classMention", "edu.uchsc.ccp.uima.mention.CCPClassMention", featOkTst);
241        casFeatCode_classMention  = (null == casFeat_classMention) ? JCas.INVALID_FEATURE_CODE : ((FeatureImpl)casFeat_classMention).getCode();
242    
243      }
244    }
245    
246    
247    
248
```
